# A compact and efficient physics-informed architecture for reconstructing and predicting complex physical fields from low accuracy sparse data

**DOI:** 10.21203/rs.3.rs-9914527/v1

**Published:** 2026-06-05

**Authors:** Runlin He, MRB Shahadat, Jiafu Wan, S M Abdullah Al Mamun, Qilin Liu, Xiaoyu Jiang, Shuolin Xiao, Zheng Li

**Affiliations:** 1 Morgan State University, Baltimore, 21251, MD, USA; 2 University of Washington, Seattle, 98195, WA, USA; 3 Johns Hopkins University, Baltimore, 21218, MD, USA; 4 Vanderbilt University, Nashville, 37240, TN, USA

**Keywords:** Physics-Informed Neural Networks (PINNs), Flow Reconstruction, Homogeneous Isotropic Turbulence, Spatiotemporal Forecasting, Super-Resolution

## Abstract

Complex physical fields are traditionally characterized through high-fidelity simulations or dense sensor networks, which provide detailed information but are often computationally or experimentally costly. Deep learning offers a promising approach for reconstructing and forecasting such fields from limited observations. However, many existing AI-based models rely on large labeled datasets and heavily parameterized architectures, making the models both data-intensive and computationally demanding to train. To overcome these limitations, we introduce compact and computationally efficient physics-informed neural networks (PINNs) designed to reduce both architectural complexity and computational overhead. We demonstrate its performance to reconstruct and forecast complex fields with limited or low accuracy multiscale spatiotemporal dynamics using laminar and turbulent flows as representative benchmarks. We first demonstrate accurate super-resolution of lid-driven cavity flows at Reynolds numbers up to Re=1000, where the PINNs reconstruct high-resolution, higher accuracy solutions (401 × 401) from coarse, low-accuracy inputs (40 × 40), reducing error significantly. Extending to three-dimensional Homogeneous Isotropic Turbulence (HIT), the model recovers fine-scale, highly accurate structures from coarse and inaccurate data, preserving key physical and statistical properties. We further show that the framework enables forecasting from limited temporal snapshots, with controlled error growth and accurate prediction of turbulent kinetic energy evolution. Finally, we investigate spatial domain extension, where the model reconstructs flow fields in previously unseen regions. The results reveal that while large-scale structures are recovered robustly, reconstruction fidelity depends strongly on the placement of sparse supervision, with physically informed sampling improving accuracy and energy consistency. The results also demonstrate that the proposed framework scales effectively to larger domains with increasingly complex flow structures. Across the tasks considered, the architecture contains approximately 1–1.5 million trainable parameters and our implementation requires a 3–5 hours of training stage for each task on a single NVIDIA A100 GPU. Together, these results demonstrate that the proposed compact architecture provides a unified and training-efficient route to physics-informed generalization across space and time, enabling the reconstruction and prediction of complex physical fields with multiscale spatiotemporal dynamics from limited observations.

## Introduction

Predicting the evolution of complex physical systems, particularly the reconstruction of continuous physical fields from incomplete observations, is a central challenge in science and engineering. It appears in atmospheric and ocean flows^1, 2^, reacting flows^3, 4^, plasma dynamics^5, 6^, biological transport^7, 8^, and energy systems such as wind farms^9, 10^. These systems are often governed by partial differential equations, but their full states are rarely observed. Measurements may be sparse, noisy, low resolution, or limited to a small part of the domain. Although high-fidelity simulations accurately resolve fine-grained field details, they remain computationally expensive when applied to complex multiscale systems. A key challenge, therefore, is how to extrapolate continuous physical fields beyond the available data while maintaining computational efficiency.

Scientific machine learning has made rapid progress recently in learning complex spatiotemporal patterns from data and accelerating physical simulations. Neural operators, including DeepONet, Fourier neural operators, and physics-informed neural operators, learn mappings between function spaces and can predict families of PDE solutions across different parameters, initial conditions, or resolutions^11–14^. Parallel to this, generative models have emerged as powerful tools for complex physical fields. Recent work has utilized adversarial neural operators, latent diffusion models, and stochastic interpolants to advance super-resolution, forecasting, and sparse reconstruction^15–18^. Collectively, these studies demonstrate that fine-scale structures and statistics must be evaluated directly rather than through pointwise error metrics alone, setting a high benchmark for any new physics-informed learning method for complex physical fields.

Physics-informed neural networks (PINNs) offer a complementary approach by embedding governing equations directly into the learning process. Instead of learning solely from labeled data, PINNs train neural networks to satisfy physical constraints such as conservation laws and PDE residuals^19, 20^. In this framework, the physical laws act as a strong regularizer that restricts the model to physically consistent solutions. This is especially critical when observations are sparse, noisy, or incomplete, as it prevents overfitting to noise and guides the predictions in regions lacking data. Furthermore, because PINNs are mesh-free, they can evaluate the solution at arbitrary continuous space-time points. Together, these features make them highly attractive for reconstruction from sparse sensors, low-resolution images, or partial-domain measurements.

However, standard coordinate-based MLP PINNs face significant limitations for true extrapolation in complex dynamical systems. They struggle with spectral shifts between training and testing domains. To improve true extrapolation under train–test spectral or parametric shifts, the learning problem can first be localized and physically structured. Space–time domain-decomposition methods, such as XPINNs^21^ and FBPINNs^22^, replace a single global coordinate MLP with coupled local networks over smaller subdomains, thereby reducing the complexity assigned to each network and allowing for local choices of architecture, loss weights, and sampling density. Furthermore, PINNs inherently suffer from spectral bias, wherein low-frequency components are learned much faster than high-frequency details^23^. While Fourier features and periodic activations mitigate this issue, they do not eliminate it entirely, though they do improve the conditioning of the learning problem^24–26^. Additionally, PINNs optimization is often hindered by gradient imbalances across the data, boundary, initial-condition, and PDE losses^27, 28^. This can be resolved by recently proposed strategies that treat the training process as an adaptive multi-objective optimization problem^27–32^. Global time-domain training can also violate temporal causality, leading to unstable long-term forecasts^33, 34^. To address this, global training can be replaced or supplemented by causal, sequential, or time-decomposed approaches. Causal PINNs weight the loss so that earlier-time dynamics are learned before later-time dynamics, while curriculum and sequence-to-sequence strategies reduce the difficulty of learning long trajectories in a single optimization problem^33, 34^. Nevertheless, these remedies do not fully remove the central challenge of extrapolation in nonlinear, multiscale, and chaotic systems: small local inaccuracies in the learned dynamics can be amplified and transferred across scales through strong nonlinear coupling, ultimately degrading global accuracy and long-term stability.

Despite these advances, improved accuracy is often obtained at the cost of increased architectural and computational complexity. Neural operators and generative models commonly require extensive high-fidelity training data, while advanced PINNs variants introduce multiple subnetworks, interface constraints, adaptive loss weights, feature embeddings, or causal training schedules^21, 22, 34, 35^. Standard pointwise PINNs also require repeated automatic differentiation over many collocation points, which becomes expensive for multidimensional and multiscale fields. For example, a recent operator–generative study on multiscale complex physical fields combined adversarial training, neural operators, and generative models for spatio-temporal super-resolution, forecasting, and sparse reconstruction^15^. The reported models contained approximately 2–4 million trainable parameters for two-dimensional super-resolution and approximately 6 million parameters for three-dimensional forecasting and sparse reconstruction. All training and inference were performed on a single NVIDIA H100 GPU, with a maximum wall-clock training time of 48 h per experiment. In the three-dimensional forecasting benchmark, the diffusion-based GenCFD baseline and the adversarial neural-operator model each contained about 6 million parameters and were trained for 48 GPU-hours, while the diffusion-based rollout required iterative denoising and took 228 s to forecast 36 time steps. These limitations motivate a lightweight physics-informed extrapolation method with a compact architecture, fewer training components, and lower computational cost.

To achieve this efficiency, we adopt a compact physics-informed architecture that integrates Fourier-feature embeddings with residual MLP connections within a standard PINN framework^19, 24, 36^. By avoiding multiple coupled subnetworks, interface constraints, and complex training schedules, this streamlined design minimizes computational overhead, substantially reduces the number of trainable parameters, and keeps overall training costs low. This design is motivated by a gap in current reconstruction and prediction for complex physical fields. Standard PINNs are often regarded as insufficient for super-resolution and forecasting of complex physical fields, even in relatively simple benchmark problems including lid-driven cavity flow, where vanilla PINNs may converge slowly, fail to reproduce reference solutions, or produce nonphysical predictions under more challenging conditions^37, 38^. In parallel, many recent studies on complex physical field super-resolution and forecast have moved toward convolutional super-resolution networks, GAN- or diffusion-based generative models, neural operators, and hybrid operator–generative architectures, often at the cost of more complicated model designs and higher computational demand^15, 39–46^. In contrast, we show that a carefully designed and relatively lightweight PINN with task-specific fine-tuning can still deliver accurate reconstruction and extrapolation for complex physical fields while retaining a purely PDE-residual-based formulation. We evaluate this lightweight architecture across three demanding extrapolation tasks: super-resolution from coarse observations, temporal forecasting beyond the observed window, and spatial-domain extension from partial-domain data.

We select turbulent flow as the benchmark system because it provides a demanding test for PDE-governed learning. Turbulence is characterized by coherent structures, intermittent fluctuations, strong nonlinear interactions, and a broad range of coupled spatio-temporal scales^47, 48^. Accurate reconstruction and prediction therefore require the model to capture both large-scale flow organization and small-scale dynamics. We therefore consider two complementary benchmark flows: homogeneous isotropic turbulence (HIT) and lid-driven cavity flow. HIT provides a canonical turbulent system with public DNS data available from the Johns Hopkins Turbulence Database (JHTDB)^49, 50^. The lid-driven cavity flow, although geometrically simple, provides a classical bounded-flow benchmark with nontrivial vortical structures and well-established reference solutions^51^. More importantly, it remains challenging for standard physics-informed learning: recent work reported that conventional PINNs failed to converge after 500,000 epochs for the lid-driven cavity problem at Re=1000 and did not reproduce the reference solution^37^. Related studies further showed that vanilla PINNs for high-Reynolds-number cavity flows can converge to unstable or nonphysical Navier–Stokes solutions rather than DNS-like solutions^38^. Together, these benchmarks allow us to test whether a relatively lightweight and simple physics-informed model can make reliable predictions beyond the observed resolution, time interval, and spatial domain.

Building on these considerations, the major contributions of our work are as follows.

1. We propose a compact and computationally efficient physics-informed neural representation for flow reconstructionand extrapolation. The model combines Fourier-feature embeddings and residual MLP connections within standard PINNs framework, and is trained using sparse observations together with governing-equation residuals. Unlike generative or computer-vision-inspired super-resolution methods that use adversarial, perceptual, or image-based sharpening components to recover high-frequency structures, the unobserved collocation points in our formulation are constrained directly by the PDE residual. This direct physics-based constraint yields a simple model structure with a small parameter count and a low training cost. This is especially important for turbulent flows, where visual similarity alone is insufficient; the model must also capture nonlinear interactions, coherent vortical structures, and the distribution of energy across scales.
2. We demonstrate accurate reconstruction and extrapolation from severely under-resolved observations in benchmark systems where standard PINNs are known to struggle. The two-dimensional lid-driven cavity problem is a classical bounded Navier–Stokes benchmark, but recent work reported that conventional PINNs failed to converge after 500,000 epochs for the cavity problem at Re=1000 and did not reproduce the reference solution^37^. Against this challenging background, our model is trained with only 40 × 40 observations and evaluated against a 401 × 401 reference solution, corresponding to only 1% of the reference grid points. In the three-dimensional homogeneous isotropic turbulence case from JHTDB, the model is trained with only 11^3^ sampled points and compared with a 32^3^ DNS reference field, corresponding to about 4.1% of the reference grid points. We further test spatial-domain extension by training the model on partial-domain observations and predicting outside the observed region. These tests show that the proposed architecture can recover physically meaningful complex fields even when the available measurements are coarse, sparse, and incomplete.
3. We show that this performance can be achieved with a substantially smaller computational footprint than recent operator-generative approaches for related field reconstruction and forecasting tasks. The two-dimensional super-resolution model contains about 1.0 million trainable parameters, and the three-dimensional forecasting model contains about 1.5 million trainable parameters. As a scale reference, a recent generative neural-operator study reported about 2–4 million trainable parameters for two-dimensional super-resolution and about 6 million parameters for three-dimensional forecasting and sparse reconstruction^15^. That study trained all models on a single NVIDIA H100 GPU, with a maximum wall-clock training time of 48 h per experiment. In our implementation, each sub-model is trained on a single NVIDIA A100 GPU in about 3–5 h. This training time could be further reduced with more advanced hardware, such as an NVIDIA H100 GPU. This cost profile provides a favorable balance among physical fidelity, extrapolation capability, and computational budget.

## Results & Discussions

**Figure 1.**
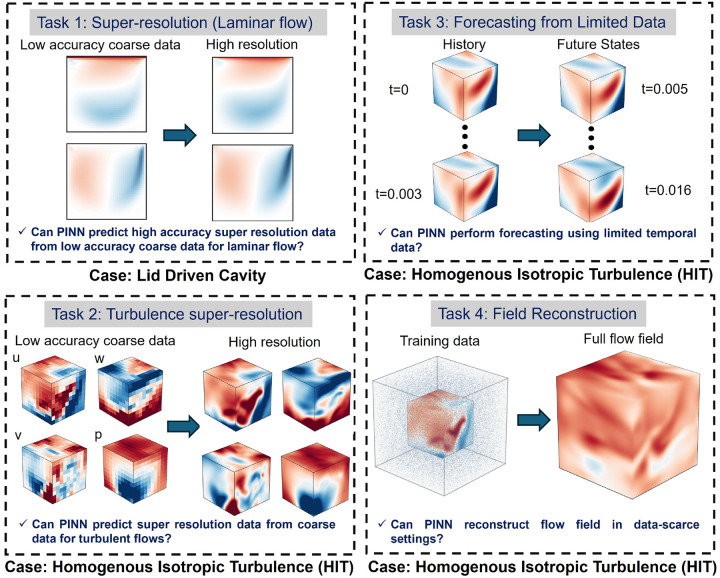
Overview of the proposed physics-informed framework. The model is evaluated across four representative tasks: (1) super-resolution of laminar flow (lid-driven cavity), where high accuracy high-resolution fields are reconstructed from coarse low accuracy inputs; (2) super-resolution of turbulent flow, demonstrating recovery of fine-scale structures in homogeneous isotropic turbulence (HIT); (3) temporal forecasting, where future flow states are predicted from limited time snapshots; and (4) field reconstruction, where the full flow field is inferred from sparse observations. Together, these tasks highlight the ability of the PINNs framework to learn a continuous, physics-consistent representation and generalize across spatial and temporal domains in data-limited settings.

We evaluate the proposed physics-informed framework across a hierarchy of flow configurations, progressing from canonical laminar benchmarks to fully developed turbulent flows (Fig. 1). This systematic approach enables a detailed assessment of the model’s ability to reconstruct, predict, and generalize across increasingly complex flow regimes.

We begin with the super-resolution of laminar flow in the classical lid-driven cavity problem at Reynolds numbers Re=100, 500, and 1000. In this setting, the model is trained using low accuracy coarse-resolution data on a 40 × 40 grid and predicts the flow field at a significantly higher resolution of 401 × 401. The coarse grid data are not obtained by downsampling a high-resolution solution, but instead originate from an independent low-resolution simulation, which inherently contains reduced accuracy. This case serves as a controlled benchmark to evaluate the accuracy of the framework to capture smooth flow structures and boundary-driven dynamics under varying levels of nonlinearity from a coarse dataset with lower accuracy.

Building on the strong performance observed in laminar flows, we extend our study to three-dimensional homogeneous isotropic turbulence (HIT), which presents a substantially more challenging test due to its multiscale, nonlinear, and chaotic nature. In this regime, we investigate three key aspects of model capability. First, we examine super-resolution, where the model predicts high-resolution flow fields on a 32 × 32 × 32 grid from low accuracy coarse inputs on a 11 × 11 × 11 grid. Second, we evaluate temporal forecasting, assessing the ability of the model to predict future flow states beyond the observed training window. Third, we consider spatial domain extension, where the model is required to reconstruct flow fields in previously unseen regions of the domain using limited supervision.

Together, these cases provide a comprehensive evaluation of the proposed framework in reconstruction, prediction, and generalization tasks. By combining sparse data with governing physical constraints, the model is shown to effectively recover flow structures, maintain physical consistency, and generalize beyond both spatial and temporal domains of the training data.

### Super-resolution of laminar flow: lid-driven cavity

We first evaluate the performance of the proposed framework on the classical lid-driven cavity problem, which serves as a canonical benchmark for incompressible laminar flow. This reference problem is widely used in computational fluid dynamics due to its simple geometry, well-defined boundary conditions, and the availability of high-fidelity reference data for different Reynolds numbers^51–54^.

**Figure 2.**
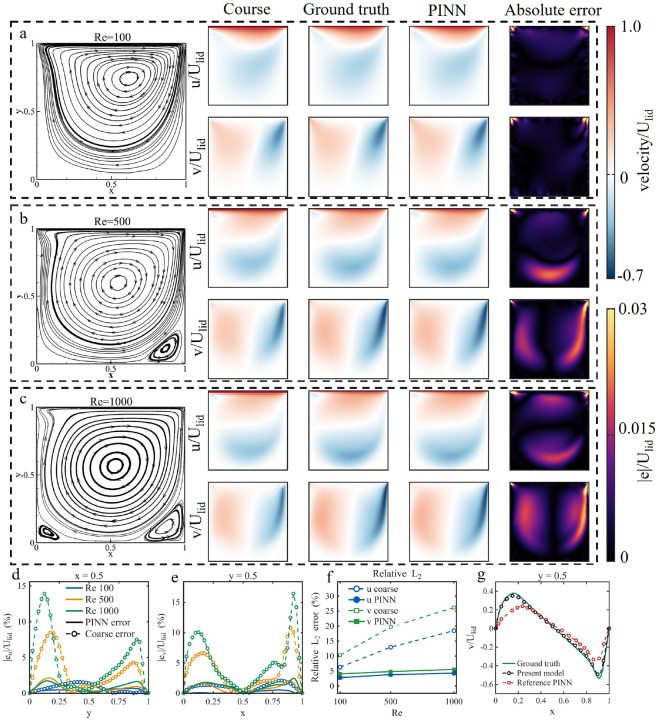
Physics-informed super-resolution of lid-driven cavity flow (Re=100,500,and1000). Comparison of coarse-resolution input (40 × 40), ground truth, and PINNs-reconstructed velocity fields (401 × 401) at different Reynolds numbers. The PINNs accurately recover both horizontal (u) and vertical (v) velocity components, capturing primary and secondary vortical structures with strong agreement with the ground truth. Absolute error fields indicate that discrepancies are localized near boundary layers and corner regions with strong gradients. Centerline error profiles further show that the PINNs consistently achieve significantly lower error than the coarse input across all Reynolds numbers. Although the error increases with Reynolds number due to enhanced flow nonlinearity, the PINNs maintain substantially improved accuracy. Our proposed PINNs framework also closely matches the reference solution across the entire domain, while significantly outperforming the baseline PINNs model reported by Karnakov et al. (2024)^37, 51^.

In this study, simulations are performed at Reynolds numbers Re=100, 500, and 1000. The model is trained using coarse-resolution data from a Lattice Boltzmann Method (LBM) simulation on a grid of 40 × 40 and is tasked with predicting the flow field at a significantly higher resolution of 401 × 401. This represents a substantial upscaling factor and provides a rigorous test of the super-resolution capability of the model. As shown in Fig. 2, the PINNs accurately reconstruct both the horizontal (u) and vertical (v) velocity components across all Reynolds numbers. The predicted fields exhibit excellent agreement with the ground truth, capturing the primary recirculation zone as well as secondary vortical structures that emerge at higher Reynolds numbers. Notably, the model preserves the smooth spatial variation of the flow and maintains consistency with the boundary conditions, particularly near the moving lid and no-slip walls.

To further quantify the super-resolution accuracy, we examine the error distribution along representative centerline locations and evaluate global error metrics across Reynolds numbers, as shown in Fig. 2. The circular markers represent the error associated with the coarse resolution input (40 × 40), while the solid lines correspond to the PINNs predictions reconstructed at a significantly higher resolution (401 × 401). All errors are computed with respect to the high-fidelity ground truth solution. A key observation from Fig. 2 (d–e) is that the PINNs consistently achieve lower error than the coarse resolution input across all Reynolds numbers and spatial locations. Despite being trained on coarse data that inherently contain numerical inaccuracies, the PINNs are able to reconstruct flow fields with substantially improved accuracy at a much finer resolution. This demonstrates that the proposed framework does not merely interpolate the coarse input, but instead learns a physically consistent representation of the flow, enabling recovery of high-resolution features that are not present in the training data. In other words, the model effectively enhances both resolution and accuracy simultaneously, which represents a significant advancement over conventional data-driven super-resolution approaches. The error profiles also reveal that discrepancies are primarily localized near regions of strong gradients, such as boundary layers and corner vortices, where the flow exhibits sharp spatial variations. These regions are inherently difficult to resolve using coarse grids, and although the PINNs significantly reduce the error, small deviations persist due to the limited information available during training. Fig. 2 (f–g) further illustrates the variation of global error metrics with Reynolds number. As expected, the relative $L_{2}$ error increases with Reynolds number for all cases, reflecting the increasing complexity and nonlinearity of the flow. At higher Reynolds numbers, thinner boundary layers and stronger convective effects make accurate reconstruction more challenging. However, it is important to note that the PINNs maintain a consistently lower error compared to the coarse resolution input across all Reynolds numbers, demonstrating its robustness in handling increasingly complex flow regimes.

The comparison in Fig. 2 (g) highlights that the proposed PINNs framework closely matches the reference solution across the entire domain, while significantly outperforming the baseline PINNs model reported by Karnakov et al. (2024)^37, 51^. The present model accurately captures both the peak and near-wall gradients, where the reference PINNs exhibit noticeable deviations. This improved agreement demonstrates the ability of the proposed approach to recover fine-scale flow features and maintain high fidelity with respect to the reference solution.

Overall, these results highlight a key capability of the proposed framework: even when trained on low-resolution and lower-accuracy data, the PINNs are able to reconstruct high-resolution flow fields with significantly improved accuracy. This ability to recover fine-scale structures and reduce error across a wide range of Reynolds numbers underscores the effectiveness of incorporating physical constraints into the learning process.

### Turbulent flow: homogeneous isotropic turbulence

To extend our proposed framework beyond laminar regimes, we next consider three-dimensional forced homogeneous isotropic turbulence (HIT), which represents a significantly more challenging test case due to its multi-scale, nonlinear, and chaotic nature^50, 55, 56^. Unlike the lid-driven cavity flow, where the dynamics are relatively smooth and boundary-driven, turbulent flows exhibit a wide range of interacting scales and strong sensitivity to perturbations, making both reconstruction and prediction inherently difficult^56, 57^.

The turbulence data used in this study are obtained from a direct numerical simulation (DNS) of three-dimensional forced homogeneous isotropic turbulence (HIT) performed on a 1024^3^ periodic grid using a pseudo-spectral solver^50^. The simulation employs a de-aliased sine phase-shift method and advances the governing equations using a second-order Adams-Bashforth scheme for nonlinear terms, while viscous effects are treated analytically via an integrating factor. Energy is continuously injected at low wavenumbers to maintain statistical stationarity, ensuring a sustained turbulent state^50, 57^. The dataset comprises 5,024 temporally resolved snapshots, corresponding to approximately five large-eddy turnover times, with stored variables including the three velocity components, pressure, and forcing terms. The spatial domain spans $[0,2\pi {]}^{3}$, with a kinematic viscosity of $v=1.85\times 10^{-4}$ and a time step of $\Delta t=2\times 10^{-4}$. Data are subsampled at intervals of 0.002, providing a temporally resolved representation of the flow evolution. The turbulence statistics are consistent with canonical HIT behavior, with a Taylor-scale Reynolds number $Re_{\lambda }\approx 433$, root-mean-square velocity $u^{\prime }\approx 0.681$, and dissipation rate ε≈0.0928. The corresponding Kolmogorov length and time scales are $\eta \approx 2.87\times 10^{-3}$ and $\tau_{\eta }\approx 0.0446$, respectively. The energy spectrum exhibits the expected inertial subrange scaling, closely following the $k^{-5/3}$ law, confirming the physical fidelity of the dataset. It is important to note that gradient quantities obtained from the database may contain small errors due to the use of spatially localized interpolation operators, as opposed to global spectral differentiation. Nevertheless, the dataset provides a high-fidelity representation of turbulent flow dynamics and serves as a reliable benchmark for evaluating physics-informed learning approaches in data-limited regimes. This dataset is publicly available in the Johns Hopkins Turbulence Database (JHTDB).

### Super-resolution in homogeneous isotropic turbulence

Having demonstrated the effectiveness of the proposed PINNs framework for laminar flows, we next extend the approach to three-dimensional homogeneous isotropic turbulence (HIT), which represents a significantly more challenging setting due to its strongly nonlinear, multi-scale nature. Unlike laminar flows, turbulent fields contain a wide range of interacting scales, making accurate reconstruction from coarse data particularly difficult, especially for small-scale structures. Notably, in lid-driven cavity flow, boundary conditions are explicitly known and provide strong constraints regardless of resolution, whereas homogeneous isotropic turbulence lacks such boundary information, making accurate reconstruction from coarse data significantly more challenging.

In this study, the model is trained on extremely coarse-resolution, low-accuracy data on an 11^3^ grid and used to reconstruct the flow field at a higher-accuracy, higher-resolution 32^3^ grid. The PINNs learn a continuous mapping from spatial coordinates (x,y,z) to the flow variables (u,v,w,p), while enforcing the governing Navier-Stokes equations through physics-based constraints.

**Figure 3.**
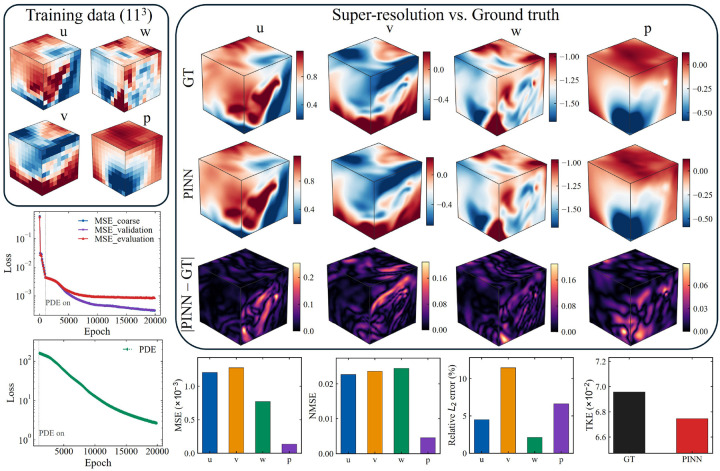
Physics-informed super-resolution of turbulent flow. The PINNs are trained on extremely coarse-resolution, inaccurate data (11^3^) and used to reconstruct the flow field at a higher resolution (32^3^). Top left: input training data for velocity components and pressure. Right: comparison between ground truth and PINNs predictions for (u,v,w,p), along with corresponding error fields. Despite low-resolution and inaccurate input, the model accurately recovers the dominant flow structures and spatial organization across all variables. Bottom: training loss convergence and comparison of data-driven and physics-based losses, demonstrating stable optimization. Quantitative metrics, including MSE, normalized MSE (NMSE), and relative $L_{2}$ error, indicate variable-dependent reconstruction accuracy, with higher errors observed in the transverse velocity component. The turbulent kinetic energy (TKE) comparison further confirms that the model preserves the flow’s overall energy content. Together, these results demonstrate that the proposed framework can reconstruct physically consistent, high-resolution, accurate turbulent fields from highly limited, inaccurate data.

Fig. 3 presents a comparison between the training data, PINNs predictions, and ground-truth solutions for all flow variables. Despite the severe under-resolution and inaccuracy of the input, the PINNs successfully reconstruct the dominant flow structures and captures the overall spatial organization across all velocity components and pressure. The predicted fields show strong qualitative agreement with the ground truth, demonstrating the model’s ability to infer missing flow information and recover coherent structures that are not explicitly present in the input data.

Quantitative evaluation further supports these observations. The error metrics, including mean squared error (MSE) and relative $L_{2}$ error, indicate that reconstruction accuracy varies across variables. The u and w velocity components exhibit relatively lower error, suggesting that the large-scale flow structures are well captured. In contrast, the v component shows higher error, reflecting its increased sensitivity to small-scale fluctuations and more complex spatial variations. The pressure field is reconstructed with comparatively high accuracy, although localized discrepancies are observed near regions of strong gradients.

Importantly, the energy-based and loss convergence plots indicate stable training behavior, with both data-driven and physics-based losses decreasing consistently during optimization. The comparison of turbulent kinetic energy (TKE) defined as

$$\text{TKE}=\frac{1}{2}\left(u^{\prime 2}+v^{\prime 2}+w^{\prime 2}\right)$$

where $u^{\prime }$, $v^{\prime }$, and $w^{\prime }$ represent the fluctuating velocity components relative to the mean flow, further confirms that the model preserves the overall energy content of the flow, despite the extremely limited resolution of the training data. TKE is a fundamental quantity in turbulence, as it characterizes the distribution of kinetic energy across scales and reflects the balance between energy production, transfer, and dissipation. Importantly, the use of turbulent kinetic energy (TKE) as an additional evaluation metric provides a physics-based measure of reconstruction quality, complementing traditional error norms and offering deeper insight into the preservation of flow dynamics.

Overall, these results demonstrate that the proposed PINNs framework can effectively perform super-resolution in turbulent flows, recovering physically consistent high-resolution fields from highly coarse, inaccurate inputs. While discrepancies persist at smaller scales, the model successfully captures the dominant flow dynamics, highlighting its potential for reconstructing complex turbulent fields in data-limited scenarios.

### Forecasting with limited temporal data

Forecasting turbulent flows remains a fundamental challenge due to the cost of acquiring high-fidelity, time-resolved volumetric data^58^. Direct numerical simulations (DNS) are computationally expensive and experimental techniques rarely provide access to complete three-dimensional flow fields. As a result, most practical settings are inherently data-limited, often restricted to a small number of snapshots from a single realization. In this data-scarce regime, physics-informed neural networks (PINNs) provide a promising framework by embedding the governing equations directly into the learning process, thereby reducing reliance on large labeled datasets and enabling generalization beyond observed data^58^. In this work, we employ a PINNs-based approach to predict future time steps in a three-dimensional forced homogeneous isotropic turbulence (HIT) using only 16 time steps from a single simulated trajectory, representing a highly constrained training scenario. Notably, training based on only 16 time steps represents an extremely limited and data-scarce training regime.

For temporal forecasting, the network is trained on a short sequence of early-time snapshots and subsequently tasked with predicting future states without access to ground-truth data at later times. In the present study, the model is trained over the interval T=0 to T=0.003 and used to predict flow fields at T=0.005,0.006,0.008,0.012,0.014, and 0.016. This setup represents a challenging extrapolation problem, as turbulent flows are inherently nonlinear, multi-scale, and sensitive to small perturbations^50, 58^.

Despite these challenges, the model is able to propagate the flow field forward in time with good accuracy over moderate forecast horizons. The predicted fields preserve the dominant coherent structures and capture the advection and deformation of large-scale features. This indicates that the model successfully learns a continuous spatio-temporal representation of the flow, rather than simply interpolating between training snapshots. The agreement with ground truth at future prediction times demonstrates that the governing physics encoded through the PDE constraint plays a crucial role in maintaining physically consistent evolution. To further assess the physical fidelity of the predictions, we examine the evolution of turbulent kinetic energy (TKE). In decaying turbulence like HIT, TKE is expected to decrease over time due to viscous dissipation. The PINNs predictions accurately reproduce this decay trend over the forecast horizon, indicating that the model preserves the global energy dynamics and remains consistent with the underlying physics.

**Figure 4.**
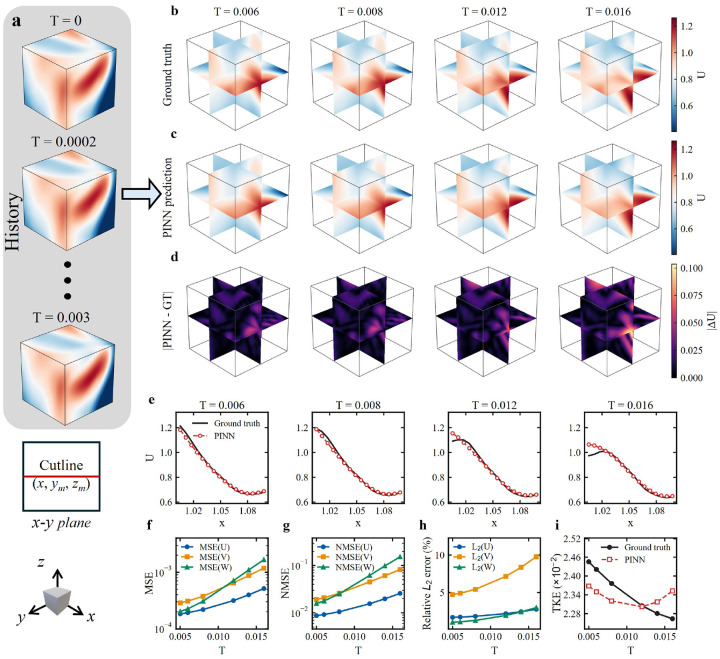
Physics-informed temporal forecasting of turbulent flow. The model is trained on a short sequence of early-time snapshots (T=0 to T=0.003) and used to predict future states at T=0.005,0.006,0.008,0.012,0.014, and 0.016 (contours at T=0.005 and 0.014 are not shown in this figure but shown in the error calculation). The PINNs accurately propagate the flow field forward in time, preserving the dominant coherent structures and their evolution with strong agreement with the ground truth. Absolute error fields show that discrepancies remain localized and increase gradually with prediction horizon, particularly in regions of strong gradients. Cutline comparisons further demonstrate close agreement between predicted and reference solutions. Error metrics (MSE, NMSE, and relative $L_{2}$) increase with time due to nonlinear error accumulation, while the predicted turbulent kinetic energy follows the expected decay trend for moderate horizons. These results demonstrate that the PINNs enable stable short to medium-term forecasting while maintaining physical consistency in a data-limited setting.

However, as the prediction extends further beyond the training window, the error begins to increase progressively, as evident from, Fig. 4 (f–h). This behavior arises from the inherent sensitivity of turbulent flows to initial conditions and small perturbations. Even minor discrepancies in the predicted velocity field can amplify over time through nonlinear interactions, leading to divergence from the true solution. Unlike deterministic time-marching solvers that explicitly enforce conservation at each time step, the PINNs represent the solution as a global function, and any error in this representation can propagate and accumulate during extrapolation. This error accumulation becomes particularly evident at longer prediction times, where deviations from physical behavior begin to emerge. In the present study, reliable predictions are obtained up to approximately four times the duration of the training window. Beyond this range, the error increases significantly, and the predicted TKE deviates from the expected decay trend, exhibiting non-physical growth. This behavior indicates that the model is no longer accurately capturing the balance between energy transfer and dissipation, and the solution begins to drift away from the true physics.

These observations highlight a fundamental limitation of long-term forecasting in turbulent flows. Due to the chaotic and multi-scale nature of turbulence, accurate prediction over extended time horizons is intrinsically difficult, regardless of the modeling approach. In the context of physics-informed neural networks, this limitation is further influenced by the relative weighting of the PDE constraint and the availability of data. While the PDE residual helps enforce physical consistency, it may not fully constrain the solution in the absence of sufficient supervision, particularly at small scales.

Overall, the forecasting results demonstrate that: (i) The proposed framework accurately predicts future time steps using highly limited training data. (ii) the prediction error increases with forecast horizon due to nonlinear error amplification, and (iii) beyond a certain temporal limit, the model may deviate from physically consistent behavior, as indicated by non-physical trends in TKE. These findings emphasize the importance of balancing model generalization with physical fidelity, and suggest that while PINNs can provide accurate short- to medium-term forecasts, caution must be exercised when extending predictions too far beyond the training window.

### Flow reconstruction from partial observations

Building on the temporal forecasting results, we next examine the spatial generalization capability of the proposed framework through a series of domain extension tests. Notably, the proposed approach does not rely on any explicit reduced-order modeling techniques, such as proper orthogonal decomposition (POD)^59–62^, or pre-defined basis functions; instead, the model learns a continuous representation of the flow field directly from sparse data under physical constraints. In contrast to temporal extrapolation, where errors accumulate due to nonlinear dynamics, spatial generalization presents a different challenge: the model must reconstruct flow structures in regions where no direct information is available, relying solely on limited supervision and governing physical constraints.

**Figure 5.**
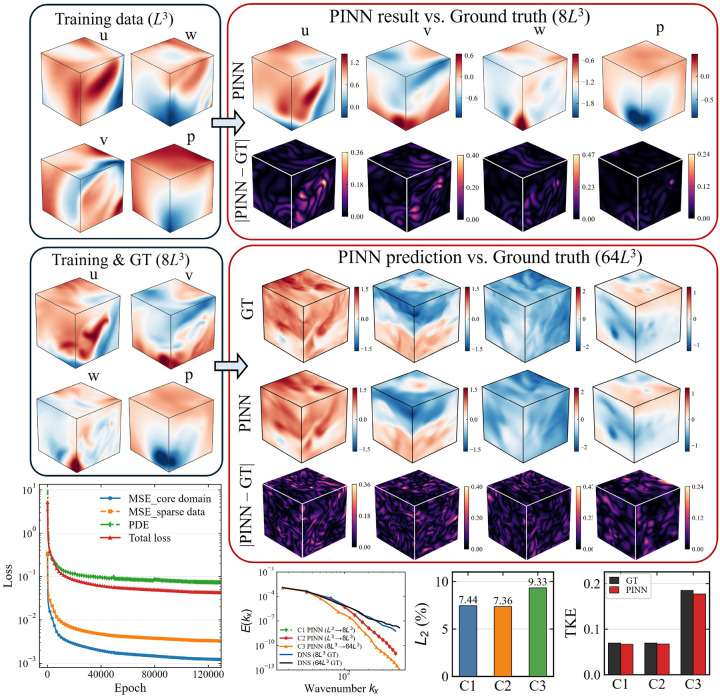
Physics-informed flow reconstruction in turbulent flow. The model is trained on a smaller domain and used to reconstruct flow fields over progressively larger domains. Top: reconstruction from $L^{3}$ to $8L^{3}$, PINNs predictions for velocity components and corresponding error fields. Bottom: further extension from $8L^{3}$ to $64L^{3}$, demonstrating scalability to larger domains. Three sampling strategies are considered: random sampling (C1), hybrid sampling with physically informed points (C2), and large-domain random sampling (C3). The PINNs accurately capture large-scale structures across all cases, while discrepancies increase in small-scale structures, in regions of strong gradients, and at larger extrapolation distances. Quantitative metrics show slightly improved accuracy for physically informed sampling (C2) and increased deviation for larger domain extension (C3), highlighting the importance of sparse data placement for maintaining spectral fidelity and physical consistency. The results also demonstrate that the proposed framework scales effectively to larger domains with increasingly complex flow structures.

We first consider a domain extension from L×L×L to 2L×2L×2L, where the model is trained using data from the smaller domain and subsequently used to reconstruct the flow in the expanded region (Fig. 5). In this setting, the extended domain contains no full-field supervision, and only sparse points are provided to guide the solution. Two sampling strategies are investigated in this study. In Case 1 (C1), 2048 points are randomly selected within the extended domain, representing a purely data-driven sparse supervision strategy. While this approach enables the model to recover the overall flow organization, noticeable discrepancies appear in regions of strong gradients and complex interactions due to the lack of targeted constraints. In Case 2 (C2), we introduce a hybrid sampling strategy, where 1024 points are randomly selected and an additional 1024 points are chosen based on regions of high Q-criterion, corresponding to dynamically important vortical structures. By incorporating supervision in these physically significant regions, the model is provided with more informative constraints, leading to improved reconstruction accuracy and better preservation of fine-scale features. This comparison highlights that both the quantity and the spatial distribution of sparse data plays a critical role in guiding the solution toward the correct physical manifold.

To further investigate the scalability of the framework, we consider a more challenging domain extension from 2L×2L×2L to 4L×4L×4L (Fig. 4). In this case (C3), the model is trained using ground-truth data from the intermediate domain (2L×2L×2L) and reconstructed the flow over a significantly larger spatial region. Sparse supervision in the extended domain is provided using 32,768 randomly distributed points, without any prior knowledge of dynamically important regions. Despite the absence of targeted sampling, the model successfully captures the global flow structures and maintains consistency with the underlying physics, although localized discrepancies remain in regions associated with high-wavenumber content.

The reconstruction performance in the extended domains is evaluated using the energy spectrum, global error metrics, and turbulent kinetic energy (TKE), as shown in Fig. 5. The energy spectra for all cases demonstrate that the PINNs accurately capture the low-wavenumber behavior, corresponding to large-scale flow structures, while deviations become more pronounced at higher wavenumbers. This is expected, as fine-scale turbulent features are more sensitive to local inaccuracies and require stronger constraints for accurate reconstruction. The energy spectra for Cases 1 and 2 show close agreement with each other, indicating that both random and hybrid sampling strategies are able to capture the large-scale energy distribution effectively. However, for the larger domain extension (Case 3), the discrepancy with respect to the DNS becomes more pronounced, particularly at higher wavenumbers, reflecting the increased difficulty of preserving fine-scale turbulent structures as the extrapolation length increases. The global error metrics further reinforce these observations. The relative $L_{2}$ error indicates that Case 2 consistently performs slightly better than Case 1, highlighting the benefit of incorporating physically informed sampling. In contrast, the larger domain extension (Case 3) exhibits a noticeable increase in error, primarily due to the greater extrapolation length and the absence of targeted supervision in dynamically important regions. This trend underscores the increasing difficulty of accurately reconstructing turbulent structures as the domain size expands, particularly for small-scale features that are more sensitive to insufficient constraints. The TKE comparison provides an additional measure of physical consistency. For Cases 1 and 2, the predicted TKE closely matches the ground truth, with deviations of only 3.72% and 3.09%, respectively, indicating that the model effectively preserves the overall energy content of the flow despite limited supervision. Case 3 shows a slightly larger deviation of 4.18%, reflecting the increased difficulty of maintaining energy consistency over larger extrapolation distances. While the model continues to capture the dominant large-scale structures, the absence of sufficient constraints in the extended domain leads to energy redistribution, particularly affecting smaller-scale dynamics.

The results of flow reconstruction demonstrate that the proposed framework effectively preserves large-scale flow physics while maintaining strong accuracy in extended domains. The findings further highlight that the placement of sparse supervision plays a critical role in improving spectral fidelity and ensuring physical consistency, particularly in capturing small-scale turbulent structures. The results also demonstrate that the proposed framework scales effectively to larger domains with increasingly complex flow structures.

Overall, these results highlight a key distinction between temporal and spatial generalization in turbulent flows. Temporal forecasting benefits from the initial conditions, allowing the model to propagate dominant structures forward in time with controlled error growth, whereas spatial extrapolation lacks such inherent constraints, making reconstruction in unseen regions significantly more challenging. This limitation is particularly evident in turbulent flows, where vortical structures are spatially intermittent and multi-scale. The results show that while random sparse supervision is sufficient to capture large-scale behavior, the inclusion of physically informative sampling points improves reconstruction accuracy, spectral fidelity, and energy consistency, especially for small-scale structures. At larger extrapolation distances, discrepancies increase, particularly at higher wavenumbers. Overall, the proposed framework demonstrates strong capability for physics-informed generalization across both temporal and spatial domains, while emphasizing the critical role of sparse data placement in achieving high-fidelity reconstruction in data-limited turbulent flows.

While the proposed framework demonstrates strong performance across multiple tasks, several directions remain for future work. Extending the approach to more complex and realistic flow configurations, including wall-bounded, shear layer, and high-Reynolds-number turbulence, will further test its generality. Improving long-term forecasting stability and enhancing scalability to larger domains are also important challenges. Addressing these aspects will broaden the applicability of physics-informed learning for real-world fluid dynamics problems.

## Methods

We employ physics-informed neural networks (PINNs) as the core modeling framework to learn continuous representations of fluid flow fields from sparse and coarse-resolution data. The core idea is to map spatial or spatio-temporal coordinates directly to flow variables while enforcing consistency with the governing Navier-Stokes equations through physics-based constraints.

**Figure 6.**
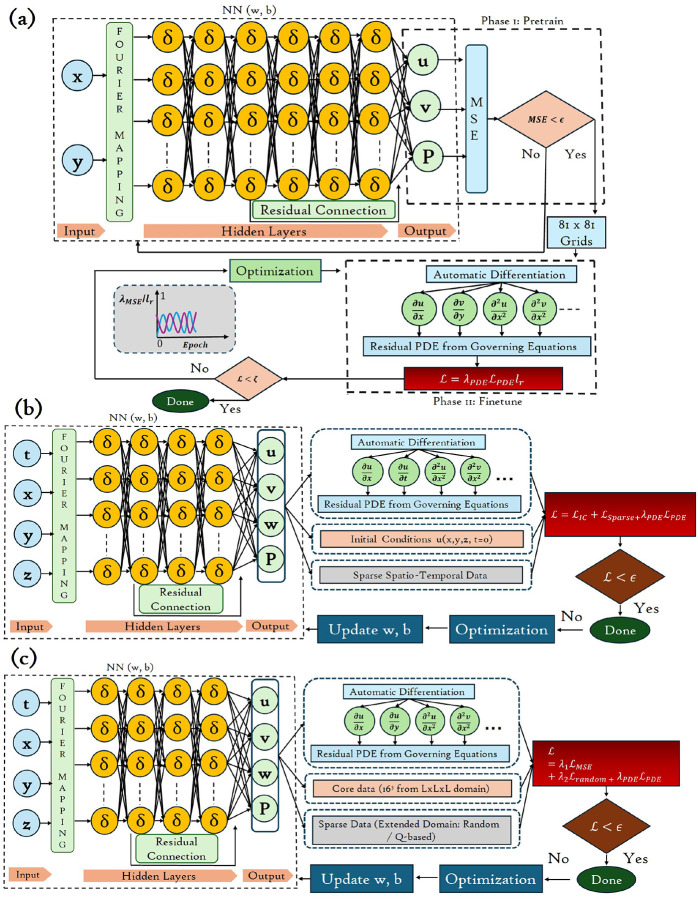
PINNs framework for multi-task flow modeling. (a) Super-resolution framework for laminar flow, where high-resolution fields are reconstructed from coarse data using a two-stage training strategy. (b) Temporal forecasting framework, incorporating initial conditions and sparse spatio-temporal data to predict future flow evolution. (c) Spatial reconstruction and domain expansion framework, where core data and sparse supervision guide the inference of flow fields in unseen regions under PDE constraints.

Although the underlying PINNs formulation remains the same, the model is adapted slightly for different tasks by modifying the input variables, supervision strategy, and loss functions (Fig. 6). This flexibility allows the same framework to be applied to super-resolution, temporal forecasting, and spatial field reconstruction across both laminar and turbulent flow regimes.

### PINNs-Based Super-Resolution Framework

We propose a physics-informed neural networks (PINNs) framework to reconstruct high-resolution velocity and pressure fields from coarse-resolution flow field data. The overall workflow is illustrated in Fig. 6. Given sparse flow information on a coarse grid, the model learns a continuous mapping from spatial coordinates (x,y) to the corresponding flow variables (u,v,p) while enforcing consistency with the governing incompressible Navier-Stokes equations.

A key challenge in flow-field super-resolution is that conventional neural networks trained solely with reconstruction losses often produce over-smoothed predictions and fail to recover localized vortical structures, sharp velocity gradients, and near-wall features. To address this limitation, we combine Fourier feature embedding, residual neural networks, and physics-guided optimization within a two-stage training framework.

The network input consists of the spatial coordinates (x,y) of the computational domain. Rather than directly using raw coordinates, we first project them into a higher-dimensional Fourier feature space using sinusoidal basis functions at multiple frequencies, (x,y)→[sin(2πBx),cos(2πBx),sin(2πBy),cos(2πBy),…], where B denotes the sampled Fourier frequencies. This transformation improves the representation of high-frequency flow structures and mitigates the spectral bias commonly observed in conventional multilayer perceptrons.

The transformed features are processed through a fully connected neural network with six hidden layers. Residual skip connections are introduced between intermediate layers to improve gradient propagation and stabilize training. Instead of learning entirely new feature representations at each layer, the network progressively learns corrections to previously extracted flow features^63^. The final network outputs the horizontal velocity, vertical velocity, and pressure fields, represented as u(x,y), v(x,y), and p(x,y), respectively. Since the model learns a continuous coordinate to field representation, it can be evaluated at arbitrary spatial resolutions during inference.

Training is performed in two stages. In the first stage, the network is pretrained using coarse-resolution flow data on a 41 × 41 grid. The predicted velocity and pressure fields are compared with reference solutions using MSE loss to learn the global flow structure. For each training coordinate $\left(x_{i},y_{i}\right)$, the predicted outputs (u,v,p) are compared against the reference values $\left(\overset{ˆ}{u},\overset{ˆ}{v},\overset{ˆ}{p}\right)$ through the MSE loss function: ${ℒ}_{\text{MSE}}=\frac{1}{N}\sum_{i=1}^{N}\left[{\left({\overset{ˆ}{u}}_{i}-u_{i}\right)}^{2}+{\left({\overset{ˆ}{v}}_{i}-v_{i}\right)}^{2}+{\left({\overset{ˆ}{p}}_{i}-p_{i}\right)}^{2}\right]$, where N denotes the number of training samples. This stage provides an informed initialization and avoids the optimization difficulties commonly encountered when PINNs are trained directly from random initialization. After convergence, the pretrained model is queried on an intermediate 81 × 81 grid to generate an initial super-resolved field. This pretraining stage provides an informed initialization before physics constraints are introduced, reducing the likelihood of convergence toward unstable or trivial solutions during subsequent PINNs optimization^64^.

In the second stage, the network is refined by enforcing physical consistency through the incompressible Navier-Stokes equations. Since the network outputs are differentiable with respect to spatial coordinates, automatic differentiation is used to compute the required first- and second-order derivatives such as $\frac{\partial u}{\partial x},\frac{\partial u}{\partial y},\frac{\partial v}{\partial x},\frac{\partial v}{\partial y},\frac{\partial^{2}u}{\partial x^{2}},\frac{\partial^{2}v}{\partial y^{2}},\ldots$ These derivatives are substituted into the momentum and continuity equations to construct the residual terms corresponding to mass and momentum conservation^65^:

(1) $$R_{u}=u\frac{\partial u}{\partial x}+v\frac{\partial u}{\partial y}+\frac{\partial p}{\partial x}-\frac{1}{Re}\left(\frac{\partial^{2}u}{\partial x^{2}}+\frac{\partial^{2}u}{\partial y^{2}}\right),$$

(2) $$R_{v}=u\frac{\partial v}{\partial x}+v\frac{\partial v}{\partial y}+\frac{\partial p}{\partial y}-\frac{1}{Re}\left(\frac{\partial^{2}v}{\partial x^{2}}+\frac{\partial^{2}v}{\partial y^{2}}\right),$$

(3) $$R_{c}=\frac{\partial u}{\partial x}+\frac{\partial v}{\partial y}.$$

The loss in physics, termed as PDE loss, is defined as the mean squared residual of these governing equations over all collocation points: ${ℒ}_{\text{PDE}}=\frac{1}{M}\sum_{k=1}^{M}\left(R_{u}^{2}+R_{v}^{2}+R_{c}^{2}\right)$, where M denotes the number of collocation points and Re is the Reynolds number^65^.

To improve optimization stability, the total loss during this stage is defined as $ℒ=\lambda_{\text{PDE}}{ℒ}_{\text{PDE}}l_{r}$, where $l_{r}$ is the learning rate and $\lambda_{PDE}$ is gradually increased during training. A small initial value prevents large residual gradients from destabilizing the pretrained solution, while larger values in later iterations enforce stronger physical consistency. The network parameters are updated iteratively using gradient-based optimization according to $\theta^{n+1}=\theta^{n}-l_{r}\nabla_{\theta }ℒ$ until the PDE residual converges, where θ denotes the trainable network parameters.

After training, the network acts as a continuous surrogate model that can be queried at arbitrary resolutions without retraining. In this study, the final reconstruction is performed on a 401 × 401 grid, resulting in the multi-resolution reconstruction pipeline 41 × 41 → 81 × 81 → 401 × 401.

For turbulent flow super-resolution, the PINNs framework is adapted to handle the increased complexity and multi-scale nature of three-dimensional turbulence. In this case, the model directly learns a mapping from spatio-temporal coordinates (x,y,z,t) to the flow variables (u,v,w,p). Unlike the laminar case, no pretraining stage is employed; instead, the network is trained in a single stage using coarse and low-accuracy data on an 11^3^ grid to reconstruct high-resolution and high-fidelity fields at 32^3^. The loss function consists of a combination of data-driven and physics-based terms, including the mean squared error (MSE) between predictions and available data, along with the Navier-Stokes PDE residual enforced over collocation points. This formulation allows the model to recover dominant turbulent structures and infer missing flow information directly from sparse and low-resolution inputs while maintaining physical consistency.

Unlike conventional interpolation techniques such as bicubic or spline interpolation, the proposed framework directly incorporates governing physics during reconstruction, enabling improved recovery of vortical structures, near-wall gradients, and pressure distributions.

### PINNs-Based Forecasting Framework

To enable temporal forecasting of turbulent flows, the PINN framework is extended to learn the spatio-temporal evolution of the flow field. In this formulation, the network maps spatio-temporal coordinates (t,x,y,z) to the corresponding flow variables (u,v,w,p), allowing it to capture both spatial structures and their temporal dynamics.

Unlike the super-resolution setting, where the objective is to reconstruct high-resolution fields from coarse spatial data, forecasting requires the model to predict future flow states beyond the observed time window. To achieve this, the network is trained using limited temporal data, consisting of an initial condition and sparse spatio-temporal observations within a short training interval. The initial condition, defined at $t=t_{0}$, provides an anchor for the temporal evolution, while sparse observations guide the learning of flow dynamics within the training window.

The governing incompressible Navier-Stokes equations are enforced throughout the spatio-temporal domain using automatic differentiation. Temporal and spatial derivatives, such as $\frac{\partial u}{\partial t},\frac{\partial u}{\partial x}$, and higher-order terms, are computed directly from the network outputs and used to construct the residuals of the momentum and continuity equations. These residuals ensure that the predicted flow fields remain physically consistent across both space and time.

The overall loss function combines contributions from the initial condition, sparse data, and PDE residual:

$$ℒ={ℒ}_{IC}+{ℒ}_{\text{sparse}}+\lambda_{PDE}{ℒ}_{PDE}.$$

Here, ${ℒ}_{IC}$ enforces agreement with the initial flow field, ${ℒ}_{\text{sparse}}$ represents the mean squared error at sparsely sampled spatio-temporal points within the training window, and ${ℒ}_{\text{PDE}}$ enforces the governing equations over collocation points.

This formulation enables the model to learn the underlying flow dynamics from limited data and propagate the solution forward in time. Once trained, the PINNs can be queried at future time instances without requiring additional supervision, allowing prediction of flow evolution beyond the training horizon while maintaining physical consistency.

### PINNs-Based Flow Reconstruction Framework

To enable spatial reconstruction and domain extension, the PINNs framework is adapted to infer flow fields in regions where no full-field data are available. In this formulation, the network learns a mapping from spatial-temporal coordinates (x,y,z,t) to the corresponding flow variables (u,v,w,p), allowing it to reconstruct the flow over an extended domain beyond the region covered by training data.

Unlike the super-resolution framework, where coarse-resolution data are available over the entire domain, and the forecasting framework, which relies on temporal evolution from an initial condition, the reconstruction setting is inherently data-sparse. The model is trained using high-fidelity data from a limited spatial region, referred to as the core domain, along with a small number of sparse observations in the extended domain.

The governing incompressible Navier-Stokes equations are enforced over the entire spatial domain using automatic differentiation. Spatial derivatives, including first- and second-order gradients, are computed directly from the network outputs and used to construct the residuals of the momentum and continuity equations. These residuals ensure that the predicted flow field remains physically consistent across both the known and unknown regions.

The overall loss function combines contributions from core data, sparse data, and physics-based constraints:

$$ℒ=\lambda_{1}{ℒ}_{\text{core}}+\lambda_{2}{ℒ}_{\text{sparse}}+\lambda_{PDE}{ℒ}_{PDE}.$$

Here, ${ℒ}_{\text{core}}$ enforces agreement with the known flow field within the core domain, ${ℒ}_{\text{sparse}}$ represents the mean squared error at sparsely sampled points in the extended domain, and ${ℒ}_{\text{PDE}}$ enforces the governing equations over collocation points throughout the domain.

Different sampling strategies are used to provide sparse supervision in the extended region, including purely random sampling and hybrid sampling based on physically relevant flow features. While random sampling captures the overall structure of the flow, physically informed sampling improves reconstruction accuracy by providing targeted information in dynamically important regions.

This formulation enables the model to propagate information from the known region into the unknown domain through the governing equations, allowing reconstruction of physically consistent flow fields without requiring full-field supervision. Once trained, the PINNs can be evaluated over the entire extended domain, effectively performing domain expansion while preserving key flow characteristics.

### Physics-Informed Training and Computational Efficiency

The proposed framework is designed to achieve accurate flow reconstruction and prediction under low accuracy, limited data and computational resources. Unlike several existing approaches that rely on large neural networks or extensive high-quality datasets, the present model leverages physics-informed constraints to reduce both model complexity and data requirements.

A key advantage of the proposed method is its reliance on physics-consistent training rather than purely data-driven learning. The model is trained using low-resolution and low-accuracy data (e.g., 40 × 40 for lid-driven cavity flow and 11^3^ for turbulent flow), while high-resolution predictions (up to 401 × 401 and 32^3^) are achieved through the enforcement of governing equations. In this setting, the PINNs effectively generate additional training information through PDE residuals, allowing the model to learn underlying physical relationships rather than simply fitting data distributions. This significantly reduces the dependence on large quantities of high-fidelity labeled data.

In contrast to recent approaches that incorporate computer vision strategies to enhance high-frequency feature representation, the present framework remains fully physics-driven. While such methods may improve visual sharpness, they often rely on learning data patterns rather than enforcing physical consistency. In our approach, all generated flow fields are constrained by the governing equations, ensuring that the learned representation reflects the underlying physics.

Another important advantage is the compactness of the model. The proposed architecture requires approximately 1–1.5 million parameters for both two-dimensional and three-dimensional tasks, which is significantly smaller than comparable models in the literature that typically require 2–6 million parameters. Despite this reduced model size, the framework achieves comparable or improved accuracy across all tasks.

The models are trained on a single NVIDIA A100 GPU, with each task requiring approximately 3–5 hours of training time. The pretraining stage for laminar super-resolution requires approximately 5 hours. This relatively short training time, combined with a smaller model size and reduced data requirements, demonstrates the computational efficiency of the proposed framework. Overall, the results highlight that accurate and physically consistent flow reconstruction and prediction can be achieved using compact models and low-quality data, without the need for large-scale datasets or highly complex architectures.

## Data Availability

The data and code will be made available upon request to the corresponding authors.

## References

[1] Vallis G. K. Atmospheric and oceanic fluid dynamics (Cambridge University Press, 2017).

[2] Kalnay E. Atmospheric modeling, data assimilation and predictability (Cambridge university press, 2003).

[3] Poinsot T. & Veynante D. Theoretical and numerical combustion (RT Edwards, Inc., 2005).

[4] Peters N. Turbulent combustion. Meas. Sci. Technol. 12, 2022–2022 (2001).

[5] Freidberg J. P. ideal MHD (Cambridge University Press, 2014).

[6] Kulsrud R. M. Plasma Physics for Astrophysics (Princeton University Press, 2005).

[7] Truskey G. A., Yuan F. & Katz D. F. Transport Phenomena in Biological Systems (Pearson Prentice Hall, 2009), 2 edn.

[8] Murray J. D. Mathematical Biology I: An Introduction (Springer, 2002), 3 edn.

[9] Stevens R. J. A. M. & Meneveau C. Flow structure and turbulence in wind farms. Annu. Rev. Fluid Mech. 49, 311–339, DOI: 10.1146/annurev-fluid-010816-060206 (2017).

[10] Porté-Agel F., Bastankhah M. & Shamsoddin S. Wind-turbine and wind-farm flows: A review. Boundary-Layer Meteorol. 174, 1–59, DOI: 10.1007/s10546-019-00473-0 (2020). 31975701 PMC6946756

[11] Lu L., Jin P., Pang G., Zhang Z. & Karniadakis G. E. Learning nonlinear operators via deeponet based on the universal approximation theorem of operators. Nat. machine intelligence 3, 218–229 (2021).

[12] Li Z. Fourier neural operator for parametric partial differential equations. arXiv preprint arXiv:2010.08895 (2020).

[13] Kovachki N. Neural operator: Learning maps between function spaces with applications to pdes. J. Mach. Learn. Res. 24, 1–97 (2023).

[14] Li Z. Physics-informed neural operator for learning partial differential equations. ACM/IMS J. Data Sci. 1, 1–27 (2024).

[15] Oommen V., Khodakarami S., Bora A., Wang Z. & Karniadakis G. E. Learning turbulent flows with generative models for super resolution and sparse flow reconstruction. Nat. Commun. 17, 3707 (2026). 41803146 10.1038/s41467-026-70145-4 PMC13103377

[16] Du P., Parikh M. H., Fan X., Liu X.-Y. & Wang J.-X. Conditional neural field latent diffusion model for generating spatiotemporal turbulence. Nat. Commun. 15, 10416 (2024). 39613755 10.1038/s41467-024-54712-1 PMC11607081

[17] Gao H., Kaltenbach S. & Koumoutsakos P. Generative learning for forecasting the dynamics of high-dimensional complex systems. Nat. Commun. 15, 8904 (2024). 39406736 10.1038/s41467-024-53165-w PMC11480383

[18] Schiødt M., Mücke N. T. & Velte C. M. Generative super-resolution of turbulent flows via stochastic interpolants. Sci. Reports (2026). 10.1038/s41598-025-34363-y PMC12859003 41491214

[19] Raissi M., Perdikaris P. & Karniadakis G. E. Physics-informed neural networks: A deep learning framework for solving forward and inverse problems involving nonlinear partial differential equations. J. Comput. physics 378, 686–707 (2019).

[20] Karniadakis G. E. Physics-informed machine learning. Nat. Rev. Phys. 3, 422–440 (2021).

[21] Jagtap A. D. & Karniadakis G. E. Extended physics-informed neural networks (xpinns): A generalized space-time domain decomposition based deep learning framework for nonlinear partial differential equations. Commun. Comput. Phys. 28 (2020).

[22] Moseley B., Markham A. & Nissen-Meyer T. Finite basis physics-informed neural networks (fbpinns): a scalable domain decomposition approach for solving differential equations: B. moseley et al. Adv. Comput. Math. 49, 62 (2023).

[23] Rahaman N. On the spectral bias of neural networks. In International conference on machine learning, 5301–5310 (PMLR, 2019).

[24] Tancik M. Fourier features let networks learn high frequency functions in low dimensional domains. Adv. neural information processing systems 33, 7537–7547 (2020).

[25] Sitzmann V., Martel J., Bergman A., Lindell D. & Wetzstein G. Implicit neural representations with periodic activation functions. Adv. neural information processing systems 33, 7462–7473 (2020).

[26] Wang S., Wang H. & Perdikaris P. On the eigenvector bias of fourier feature networks: From regression to solving multi-scale pdes with physics-informed neural networks. Comput. Methods Appl. Mech. Eng. 384, 113938 (2021).

[27] Wang S., Teng Y. & Perdikaris P. Understanding and mitigating gradient flow pathologies in physics-informed neural networks. SIAM J. on Sci. Comput. 43, A3055–A3081 (2021).

[28] Wang S., Yu X. & Perdikaris P. When and why pinns fail to train: A neural tangent kernel perspective. J. Comput. Phys. 449, 110768 (2022).

[29] McClenny L. D. & Braga-Neto U. M. Self-adaptive physics-informed neural networks. J. Comput. Phys. 474, 111722 (2023).

[30] Jagtap A. D., Kawaguchi K. & Karniadakis G. E. Adaptive activation functions accelerate convergence in deep and physics-informed neural networks. J. Comput. Phys. 404, 109136 (2020). 10.1098/rspa.2020.0334 PMC7426042 32831616

[31] Wu C., Zhu M., Tan Q., Kartha Y. & Lu L. A comprehensive study of non-adaptive and residual-based adaptive sampling for physics-informed neural networks. Comput. Methods Appl. Mech. Eng. 403, 115671 (2023).

[32] Yu J., Lu L., Meng X. & Karniadakis G. E. Gradient-enhanced physics-informed neural networks for forward and inverse pde problems. Comput. Methods Appl. Mech. Eng. 393, 114823 (2022).

[33] Krishnapriyan A., Gholami A., Zhe S., Kirby R. & Mahoney M. Characterizing possible failure modes in physics-informed neural networks. Adv. neural information processing systems 34, 26548–26560 (2021).

[34] Wang S., Sankaran S. & Perdikaris P. Respecting causality for training physics-informed neural networks. Comput. Methods Appl. Mech. Eng. 421, 116813 (2024).

[35] Shukla K., Jagtap A. D. & Karniadakis G. E. Parallel physics-informed neural networks via domain decomposition. J. Comput. Phys. 447, 110683, DOI: 10.1016/j.jcp.2021.110683 (2021).

[36] He K., Zhang X., Ren S. & Sun J. Deep residual learning for image recognition. In Proceedings of the IEEE Conference on Computer Vision and Pattern Recognition, 770–778 (2016).

[37] Karnakov P., Litvinov S. & Koumoutsakos P. Solving inverse problems in physics by optimizing a discrete loss: Fast and accurate learning without neural networks. PNAS nexus 3, pgae005 (2024). 38250513 10.1093/pnasnexus/pgae005 PMC10799659

[38] Wang Z., Meng X., Jiang X., Xiang H. & Karniadakis G. E. Solution multiplicity and effects of data and eddy viscosity on Navier–Stokes solutions inferred by physics-informed neural networks (2023). 2309.06010.

[39] Fukami K., Fukagata K. & Taira K. Super-resolution reconstruction of turbulent flows with machine learning. J. Fluid Mech. 870, 106–120, DOI: 10.1017/jfm.2019.238 (2019).

[40] Fukami K., Fukagata K. & Taira K. Machine-learning-based spatio-temporal super resolution reconstruction of turbulent flows. J. Fluid Mech. 909, A9, DOI: 10.1017/jfm.2020.948 (2021).

[41] Liu B., Tang J., Huang H. & Lu X.-Y. Deep learning methods for super-resolution reconstruction of turbulent flows. Phys. Fluids 32, 025105, DOI: 10.1063/1.5140772 (2020).

[42] Deng Z., He C., Liu Y. & Kim K. C. Super-resolution reconstruction of turbulent velocity fields using a generative adversarial network-based artificial intelligence framework. Phys. Fluids 31, 125111, DOI: 10.1063/1.5127031 (2019).

[43] Gao H., Sun L. & Wang J.-X. Super-resolution and denoising of fluid flow using physics-informed convolutional neural networks without high-resolution labels. Phys. Fluids 33, 073603, DOI: 10.1063/5.0054312 (2021).

[44] Li Z. Fourier neural operator for parametric partial differential equations. In International Conference on Learning Representations (2021). PMC13460308 42583553

[45] Li Z., Peng W., Yuan Z. & Wang J. Fourier neural operator approach to large eddy simulation of three-dimensional turbulence. Theor. Appl. Mech. Lett. 12, 100389, DOI: 10.1016/j.taml.2022.100389 (2022).

[46] Li Z., Peng W., Yuan Z. & Wang J. Long-term predictions of turbulence by implicit U-Net enhanced fourier neural operator. Phys. Fluids 35, 075145, DOI: 10.1063/5.0158830 (2023).

[47] Pope S. B. Turbulent Flows (Cambridge University Press, 2000).

[48] Frisch U. Turbulence: The Legacy of A. N. Kolmogorov (Cambridge University Press, 1995).

[49] Perlman E., Burns R., Li Y. & Meneveau C. Data exploration of turbulence simulations using a database cluster. In Proceedings of the 2007 ACM/IEEE Conference on Supercomputing, 1–11 (2007).

[50] Li Y. A public turbulence database cluster and applications to study lagrangian evolution of velocity increments in turbulence. J. Turbul. N31 (2008).

[51] Ghia U., Ghia K. N. & Shin C. High-re solutions for incompressible flow using the navier-stokes equations and a multigrid method. J. computational physics 48, 387–411 (1982).

[52] Li Z., Yang M. & Zhang Y. Hybrid lattice boltzmann and finite volume methods for fluid flow problems. Int. J. for Multiscale Comput. Eng. 12 (2014).

[53] Li Z., Yang M. & Zhang Y. A hybrid lattice boltzmann and monte carlo method for natural convection simulation. Int. J. for Multiscale Comput. Eng. 13 (2015).

[54] Rao C., Sun H. & Liu Y. Physics-informed deep learning for incompressible laminar flows. Theor. Appl. Mech. Lett. 10, 207–212 (2020).

[55] Cao N., Chen S. & Doolen G. D. Statistics and structures of pressure in isotropic turbulence. Phys. Fluids 11, 2235–2250 (1999).

[56] Yu H. Studying lagrangian dynamics of turbulence using on-demand fluid particle tracking in a public turbulence database. J. Turbul. N12 (2012).

[57] Patterson G. & Orszag S. A. Spectral calculations of isotropic turbulence: Efficient removal of aliasing interactions. Phys. Fluids 14, 2538 (1971).

[58] Oommen V., Khodakarami S., Bora A., Wang Z. & Karniadakis G. E. Learning turbulent flows with generative models: Super-resolution, forecasting, and sparse flow reconstruction. arXiv preprint arXiv:2509.08752 (2025). 10.1038/s41467-026-70145-4 PMC13103377 41803146

[59] Callaham J. L., Maeda K. & Brunton S. L. Robust flow reconstruction from limited measurements via sparse representation. Phys. Rev. Fluids 4, 103907 (2019).

[60] Zhao W. Efficient flow field reconstruction without explicit regularization terms: A snapshot-weighted approach for aerospace applications. Aerosp. Sci. Technol. 112308 (2026).

[61] Jiang W. Data-driven physical fields reconstruction of supercritical-pressure flow in regenerative cooling channel using pod-ae reduced-order model. Int. J. Heat Mass Transf. 217, 124699 (2023).

[62] Jiang G. Online reconstruction of 3d temperature field fused with pod-based reduced order approach and sparse sensor data. Int. J. Therm. Sci. 175, 107489 (2022).

[63] Lu L., Jin P. & Karniadakis G. E. Deeponet: Learning nonlinear operators for identifying differential equations based on the universal approximation theorem of operators. arXiv preprint arXiv:1910.03193 (2019).

[64] Guo J., Yao Y., Wang H. & Gu T. Pre-training strategy for solving evolution equations based on physics-informed neural networks. J. Comput. Phys. 489, 112258 (2023).

[65] Lu T. Gradient driven physics informed neural networks for conduction heat transfer and incompressible laminar flow. J. Comput. Nonlinear Dyn. 1–18 (2025). 10.1115/1.4070545 PMC12757984 41487151

